# Risk factors analysis of right ventricular-arterial uncoupling in patients having acute heart failure with preserved ejection fraction accompanied by coronary artery disease

**DOI:** 10.1186/s12872-026-05684-1

**Published:** 2026-03-06

**Authors:** Hongdan Jia, Fang Zhang, Zihou Liu, Jun Zhao, Li Liu, Lixiang Ma

**Affiliations:** 1 Department of Cardiology, First Hospital of Qinhuangdao, 258 Culture Road, Harbour District, Qinhuangdao, Hebei 066000 China; 2 Department of Ultrasound Diagnosis, First Hospital of Qinhuangdao, 258 Culture Road, Harbour District, Qinhuangdao, Hebei 066000 China; 3 Department of Cardiovascular Surgery, Chest Hospital, Tianjin University, No. 261 Taierzhuang South Road, Jinnan District, Tianjin, 300222 China

**Keywords:** Heart failure with preserved ejection fraction, Coronary artery disease, Right ventricular–arterial coupling, Risk factor, Interleukin-6

## Abstract

**Background:**

Right ventricular (RV)-arterial uncoupling is highly prevalent in patients having acute heart failure with preserved ejection fraction (HFpEF) accompanied by coronary artery disease (CAD), and it serves as a strong predictor of adverse outcomes. This study aimed to analyze the risk factors of RV-arterial uncoupling in order to explore novel therapeutic targets in acute HFpEF patients with CAD.

**Methods:**

This prospective study included 456 consecutive acute HFpEF patients with CAD. The patients were divided into RV-arterial uncoupling and coupling groups based on the optimal cutoff value, determining from a receiver operating characteristic (ROC) curve of tricuspid annular plane systolic excursion to pulmonary artery systolic pressure (TAPSE/PASP). Independent risk factors for RV-arterial uncoupling were identified using logistic regression analysis, and combined diagnosis of RV-arterial uncoupling was constructed. A ROC curve was then drawn to evaluate the influencing factors and the effectiveness of combined diagnosis.

**Results:**

In acute HFpEF patients with CAD, a TAPSE/PASP ratio of ≤ 0.43 provided good accuracy in identifying patients with RV-arterial uncoupling, with an area under the curve (AUC), 0.701, sensitivity of 58.6%, and specificity of 77.7%. Multivariable logistic regression analysis revealed that E/e´ (early diastolic mitral inflow velocity/early diastolic septal mitral annular tissue velocity), the internal diameter of the inferior vena cava (IVC), history of hypertension, atrial fibrillation, postoperative coronary artery bypass grafting (CABG) and interleukin-6 levels were the independent risk factors for RV-arterial uncoupling in acute HFpEF patients with CAD. Furthermore, left ventricular ejection fraction (LVEF), body mass index (BMI), red blood cell distribution width-standard deviation (RDW-SD), and serum albumin levels were protective factors for RV-arterial uncoupling (*P* < 0.05). A combination of 9 common indexes, including E/e´, LVEF, IVC, hypertensive history, atrial fibrillation, postoperative CABG, BMI, RDW-SD, and serum albumin, was constructed and this common index was combined with interleukin-6 again to draw a ROC curve together with interleukin-6. The AUCs for the common index, interleukin-6, and the common index combined with interleukin-6 in diagnosing RV-arterial uncoupling were 0.862 (95% *CI*, 0.817–0.899), 0.944 (95% *CI*, 0.917–0.965) and 0.980 (95% *CI*, 0.957–0.993), respectively, with *P* < 0.001. Pairwise comparisons of these three evaluation methods showed *P* values all < 0.001, with the common index combined with interleukin-6 demonstrating the highest diagnostic efficacy for RV-arterial uncoupling, achieving a sensitivity of 94.8%, a specificity of 91.6%, and an accuracy of 92.9%.

**Conclusions:**

In acute HFpEF patients with CAD, E/e´, IVC, a history of hypertension, atrial fibrillation, CABG, and interleukin-6 are associated with an increased risk of RV-arterial uncoupling, whereas LVEF, BMI, RDW-SD, and serum albumin levels serve as positive factors. Interleukin-6 plays a significant role in RV-arterial uncoupling, and its clinical value is worthy of attention.

## Introduction

The proportion of patients with heart failure (HF) with preserved ejection fraction (HFpEF) continues to rise within the overall HF population [1, 2]. The 2023 ACC Expert Consensus Decision Pathway on Management of HFpEF re-emphasizes that the current challenge for HFpEF treatment lies in the identification and management of HFpEF comorbidities, including hypertension, diabetes, obesity, atrial fibrillation, coronary heart disease, chronic kidney disease, and obstructive sleep apnea [3]. In 2021, JAMA Cardiology reported that 91% of patients with HFpEF exhibit the symptoms of epicardial Coronary Artery Disease (CAD), Coronary Microvascular Dysfunction (CMD), or both [4]. Such patients may not be able to explain the progression and deterioration of their conditions simply and singularly with the general characteristics of myocardial ischemia.

For many years, HFpEF was considered an isolated left ventricular (LV) condition. However, numerous previous studies have shown that right ventricular (RV) dysfunction and increased RV afterload are major contributors to the pathogenesis of HFpEF [5]. As a measure of RV function and its afterload matching, the role of RV-arterial coupling in HFpEF has attracted increasing attention [6]. The gold standard for evaluating RV-arterial coupling [7] is the ratio of RV pressure-volume loop-derived RV end-systolic elasticity to pulmonary artery vascular elasticity obtained through invasive right heart catheterization (end-systolic elastance/arterial elastance, Ees/Ea). However, this gold standard RV pressure-volume curve is obtained by invasive methods, and there are multiple risks in operating HF patients. Currently, most studies use the ratio of Tricuspid Annular Plane Systolic Excursion (TAPSE) to Pulmonary Artery Systolic Pressure (PASP), measured by echocardiography, as a gold standard best noninvasive surrogate in evaluating RV-arterial coupling in HFpEF [6, 8, 9], and RV-arterial coupling based on the TAPSE/PASP ratio has been proven to be a powerful predictor of the prognosis of HFpEF [10]. The TAPSE/PASP ratio may differ based on different study populations and different endpoints. As such, RV-arterial uncoupling was defined as TAPSE/PASP ratio<0.36, TAPSE/PASP ratio<0.48 and TAPSE/PASP ratio ≤ 0.40 in the prior studies [6, 8, 9], starting from a cutoff for poor prognosis. The physiological significance is that the RV function is insufficient to overcome its increased afterload which develops a poor prognosis. Recent studies have identified RV-arterial coupling as a potential therapeutic target for HFpEF [11].

The primary objective of this study was to identify the independent risk factors associated with RV-arterial uncoupling in patients presenting with acute HFpEF accompanied by CAD. The secondary objective was to establish and evaluate a combined diagnostic model integrating clinical, laboratory, and echocardiographic parameters for predicting RV-arterial uncoupling.

## Methods

### Patient population

In this study, 456 patients diagnosed with acute HFpEF with CAD were consecutively enrolled from the Department of Cardiology of the First Hospital of Qinhuangdao, China, from May 2021 to May 2022. The inclusion criteria were as follows: evidence of CAD (defined as at least one coronary artery showing ≥ 75% luminal stenosis, coronary artery bypass grafting on coronary angiography, or coronary artery CT angiography), or a past medical history of myocardial infarction, percutaneous coronary intervention, LVEF ≥ 50% on echocardiography, HFA-PEFF score ≥ 5 [12], and a diagnosis of acute HF [13]. Patients with the following conditions were excluded: primary valvular heart disease, constrictive pericarditis, hypertrophic cardiomyopathy, restrictive cardiomyopathy, chronic obstructive pulmonary disease, pulmonary hypertension, chronic thromboembolic pulmonary hypertension; acute coronary syndrome or coronary revascularization within 4 weeks prior to admission; chronic kidney disease stage 5; a history of hematologic disease or malignancy; age < 18 years old; life expectancy < 6 months; a history of heart transplantation, or in-hospital death. 

### Ethical statement

The study protocol was approved by the Ethics Committee of First Hospital of Qinhuangdao, China and adhered to the Declaration of Helsinki.

### Clinical data

This study is a prospective, single-centered, observational study that collected patients’ demographic data, clinical characteristics, laboratory tests, echocardiographic results, coronary angiography or coronary CT angiography results during hospitalization, as well as relevant past medical history and medication information. An experienced echocardiographer, who was blinded to the patient data, performed comprehensive echocardiographic examinations according to American Society of Echocardiography guidelines. The left atrial anterior-posterior dimension (LAD) was assessed via 2D measures. LV internal dimension diastole (LVIDD) was measured at end-diastole using 2D-guided linear measurements. LV volumes were calculated according to the modified biplane Simpson’s rule from apical 2- and 4-chamber views. LV volume measurements at end-diastole and end-systole were used to calculate the LVEF. Stroke volume (SV) was calculated using LV end-diastolic volume and LV end-systolic volume, and Cardiac output (CO) was calculated using SV and heart rate. The right atrium (RA) diameter at the base was estimated using conventional two-dimensional echocardiography from a right-heart apical four-chamber view. RV systolic function was assessed based on TAPSE. Right atrial pressure was estimated based on the diameter and collapsibility of the inferior vena cava (IVC) during inspiration from the subcostal view, with the following criteria: 3 mmHg for IVC < 2.1 cm with > 50% collapse, 15 mmHg for IVC > 2.1 cm with < 50% collapse, and 8 mmHg (range 5–10 mmHg) for IVC and collapse not fitting in this paradigm. Peak tricuspid regurgitation (TR) velocity was measured, and PASP was estimated to be 4× (peak TR velocity)^2^ + RA pressure. Early diastolic mitral inflow velocity (E), early diastolic septal mitral annular tissue velocity (eʹ), and the ratio of E/e′were evaluated using pulse wave Doppler and tissue Doppler imaging. Interventricular septum (IVS) thickness and LV posterior wall thickness (PWT) were measured at end-diastole using a linear method. LV mass (LVM) and relative wall thickness (RWT) were calculated as follows [14]:

$$\mathrm{L}\mathrm{V}\mathrm{M}={0.8\times(1.04\times[(\mathrm{I}\mathrm{V}\mathrm{S}+\mathrm{L}\mathrm{V}\mathrm{I}\mathrm{D}\mathrm{D}\hspace{0.17em}+\mathrm{P}\mathrm{W}\mathrm{T})}^{3}-{\mathrm{L}\mathrm{V}\mathrm{I}\mathrm{D}\mathrm{D}}^{3}]+0.6\mathrm{g}\mathrm{r}\mathrm{a}\mathrm{m}\mathrm{s}$$

$$\mathrm{R}\mathrm{W}\mathrm{T}=(2\times\mathrm{P}\mathrm{W}\mathrm{T})/\left(\mathrm{L}\mathrm{V}\mathrm{I}\mathrm{D}\mathrm{D}\right)$$

To evaluate LV morphology, the LV mass index (LVMI) normalized to body surface area (LVM divided by body surface area and RWT) was calculated.

Study Endpoint and Follow-UpThe primary endpoint was a composite of all-cause deaths, recurrent ischemic events, and HF hospitalizations. Recurrent ischemic events were defined as acute coronary syndrome(ACS)occurring after discharge. Patients were followed up via telephone consultations. The follow-up period extended from the day of the first admission until the first occurrence of the primary endpoint or the end of the study.

### Definition of variables and common index

To assess the collective influence of significant predictors, a “common index” was created. This index represents the predicted probability derived from a multivariate logistic regression model that included all identified independent clinical/laboratory/echocardiographic factors of RV-arterial uncoupling, deliberately excluding interleukin-6 to allow for its separate analysis. Given its pivotal role in RV and pulmonary artery remodeling—driving inflammation and oxidative stress, interleukin-6 was treated as a standalone biomarker. This approach allows for a direct assessment of its independent impact on RV-arterial uncoupling relative to the common index. Common index combined with interleukin-6 was constructed by adding interleukin-6 as a variable to common index to obtain the predicted probability derived from a multivariate logistic regression model.

### Sample size calculation

Sample size calculation was performed based on the logistic regression analysis. Assuming a two-sided significance level (α) of 0.05 and a statistical power (1–β) of 85%, when the independent variable increases from its mean to mean plus one standard deviation, outcome variable = 1 (representing RV-arterial uncoupling) was assumed to increase from 0.4 to 0.5, corresponding to an odds ratio (OR) of 1.5. Under these assumptions, the minimum required sample size was 227. Considering an anticipated dropout rate of 10%, the adjusted sample size was calculated as 253. In the present study, a total of 404 patients were finally included, which exceeds the estimated requirement and ensures adequate statistical power for the analyses.

### Statistical analysis

SPSS 22.0 (SPSS Inc., Armonk, NY, USA) and MedCalc 20.1 (MedCalc Software Ltd., Acacialaan 22, 8400 Ostend, Belgium) were employed for statistical analysis. The Kolmogorov-Smirnov test, histogram, and Q-Q plot were used to evaluate whether the continuous variables followed a normal distribution. Measurement data are expressed by the mean±standard deviation (* ±s*) or median (interquartile range [IQR]) M (Q1, Q3). The independent sample T test was used for comparing normally distributed measurement data between groups, while the Wilcoxon rank sum test was used for comparing data that did not follow a normal distribution. Counting data is expressed as a frequency and percentage, while the *χ2* test was used for comparisons between groups. The ROC curve and AUC with 95% CI were used to evaluate the discriminatory and predictive ability of TAPSE/PASP for the primary endpoint, and the optimal cut-off value of TAPSE/PASP at the occurrence of the primary endpoint was determined according to the Youden’s index. Based on this cut-off value, RV-arterial uncoupling was defined as TAPSE/PASP ≤ cut-off value, and patients were divided into an RV-arterial coupling group and an RV-arterial uncoupling group. Risk factors for RV-arterial uncoupling were identified using univariate and multivariate logistic regression with a bidirectional stepwise selection method (*P* < 0.05 for both entry and removal). The results of multivariate logistic regression were evaluated in terms of area under the ROC curve, sensitivity, specificity, etc. The significance of AUC with 95% CI was assessed by DeLong and Binomial exact methods to compare diagnostic performances between ROC curves. *P* < 0.05 (two-tailed) was considered statistically significant.

## Results

Of the 456 patients consecutively included in this study, 35 patients were diagnosed with ACS, 11 patients had missing PASP data, and 6 patients were excluded due to in-hospital death. Finally, 404 patients were included in the study. At a median follow-up of 290 days (IQR 174, 349), a total of 157 patients (38.86%) reached the primary endpoint event.

### Description of the TAPSE/PASP distribution

**Fig. 1:**
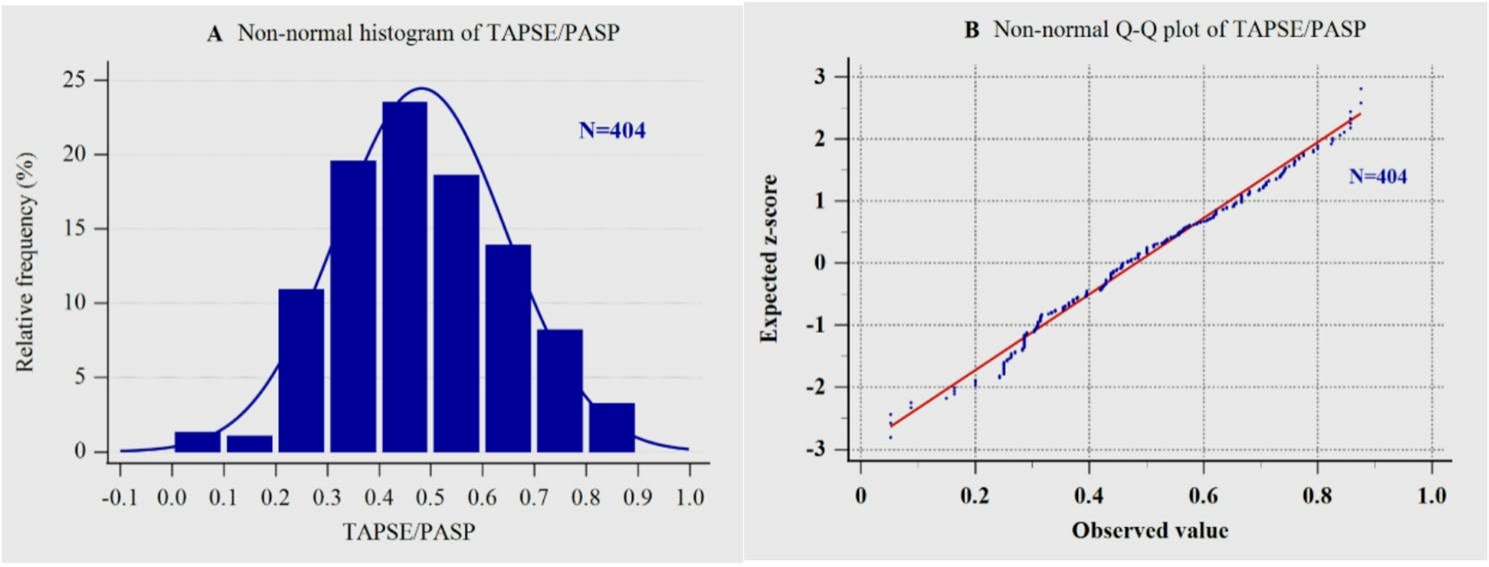
Non-normal distribution, both histogram (**A**) and Q-Q plot (**B**), of TAPSE/PASP in acute HFpEF patients with CAD. CAD, Coronary artery disease; HFpEF, Heart failure with preserved ejection fraction; TAPSE/PASP, Tricuspid annular plane systolic excursion to pulmonary artery systolic pressure

Figure 1 A shows the distribution of TAPSE/PASP in acute HFpEF patients with CAD.The Q-Q plot indicates that the TAPSE/PASP ratios are not normally distributed (Shapiro-Wilk test, P = 0.002; Fig. 1B).

### Prognostic value of RV-Arterial uncoupling

**Fig. 2:**
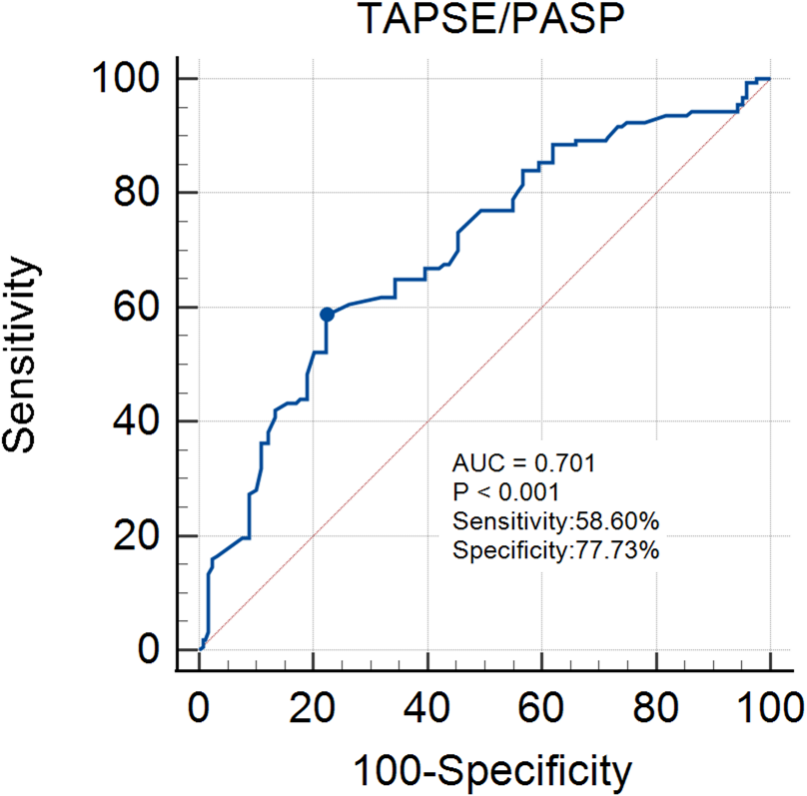
ROC curve of TAPSE/PASP ratio for predicting the primary endpoint in acute HFpEF patients with CAD. The dot represents TAPSE/PASP = 0.43. AUC, Area under the curve; CAD, Coronary artery disease; HFpEF, Heart failure with preserved ejection fraction; ROC, Receiver operating characteristic; TAPSE/PASP, Tricuspid annular plane systolic excursion to pulmonary artery systolic pressure

The ROC curve was plotted according to the TAPSE/PASP ratio and the primary endpoint, and the TAPSE/PASP ratio that corresponded to the largest AUC was determined using the Youden’s index to obtain the optimal cut-off value. The results showed that, among the 404 acute HFpEF patients with CAD, when the Youden’s index is 0.363, a TAPSE/PASP ratio of 0.43 is the optimal cut-off value for predicting the occurrence of the primary endpoint, with a sensitivity of 58.6%, a specificity of 77.7%, and a maximum AUC of 0.701 (95% CI, 0.654–0.745, *P* < 0.001), as shown in Fig. 2.

### Grouping and baseline characteristics

**Fig. 3:**
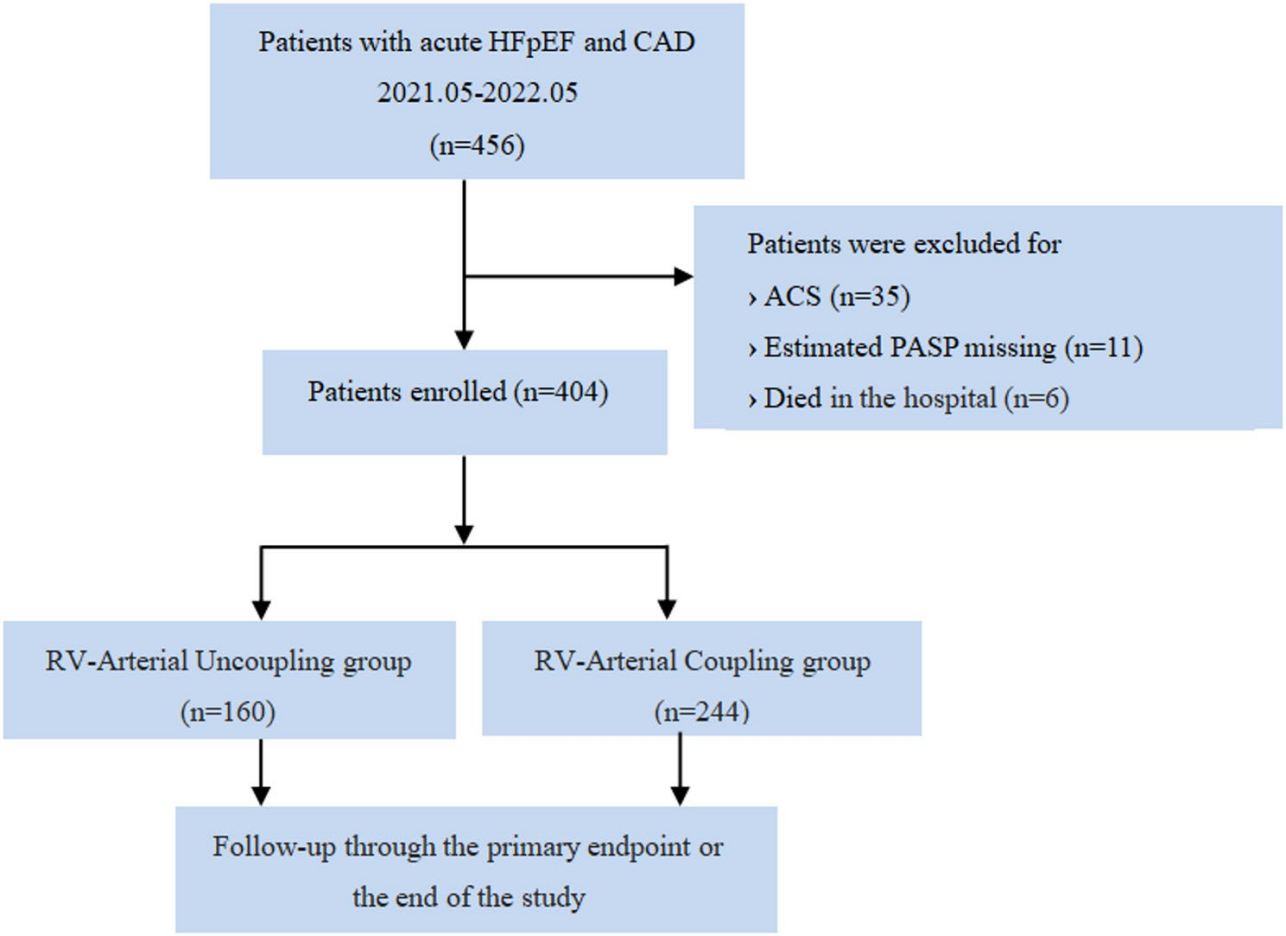
Flowchart of the study. ACS, Acute coronary syndrome; CAD, Coronary artery disease; HFpEF, Heart failure with preserved ejection fraction; PASP, Pulmonary artery systolic pressure; RV, Right ventricular

A TAPSE/PASP ratio ≤ 0.43 was defined as RV-arterial uncoupling, with the associated patients divided into an RV-arterial coupling group (TAPSE/PASP ratio > 0.43, 244 cases) and an RV-arterial uncoupling group (TAPSE/PASP ratio ≤ 0.43, 160 cases) (Fig. 3), with a median age of 66 (55–72) years. Compared with patients in the RV-arterial coupling group, patients in the RV-arterial uncoupling group were significantly older (69 [57–73] vs. 64 [55–72], *p* = 0.004). The RV-arterial uncoupling group also had a lower body mass index and a lower systolic blood pressure (23.4 ± 4.0 vs. 24.9 ± 4.1, *p* < 0.001 and 132 [116–144] vs. 139 [124–153], *p* < 0.001, respectively), and a smaller body surface area (1.7 [1.5–1.8] vs. 1.8 [1.6–1.9], *p* < 0.001). In addition, more patients in the RV-arterial uncoupling group suffered from hypertension (100 [62.50%] vs. 125 [51.23%], *p* = 0.026), atrial fibrillation (60 [37.50%] vs. 62 [25.41%], *p* = 0.010), and chronic kidney disease (59 [36.88%] vs. 65 [26.86%], *p* = 0.033), while fewer patients suffered from stroke (26 [16.25%] vs. 82 [33.61%], *p* < 0.001). In the RV-arterial uncoupling group, the proportion of patients who had undergone prior PCI therapy was small (27 [16.88%] vs. 65 [26.64%], *p* = 0.022), while the proportion of patients after CABG was relatively large (21 [13.13%] vs. 8 [3.28%], *p* < 0.001). Patients in the RV-arterial uncoupling group were more likely to use diuretics (140 [87.50%] vs. 192 [78.69%], *p* = 0.024). Additionally, in the RV-arterial uncoupling group, Triglyceride(TG)was lower (1.1 [0.9–1.8] vs. 1.4 [1.1–1.9], *p* < 0.001), while red blood cell distribution width-standard deviation (RDW-SD༉, cTnI, and NT-proBNP were relatively higher (45.7 [43.1–47.9] vs. 44.1 [41.6–47.2], *p* = 0.001; 0.03 [0.02–0.12] vs. 0.02 [0.02–0.07], *p* = 0.008 and 6290 [2309–10955] vs. 1810 [1189–4020], *p* < 0.001, respectively). Serum albumin was lower (36.8 [34.3–39.5] vs. 39.0 [35.2–41.4], *p* < 0.001), and interleukin-6 was higher (28.2 ± 8.2 vs. 11.7 ± 8.0, *p* < 0.001). E/eʹ was larger according to the data measured by echocardiography (26.1 [18.4–36.0] vs. 18.5 [15.9–24.9], *p* < 0.001); the left atrial diameter was relatively larger (4.6 [4.1–5.1] vs. 4.5 [4.1–4.9], *p* = 0.010); the LVEF was relatively lower (57 [51–63] vs. 61 [52–67], *p* = 0.016); the IVC and right atrium diameter (RAD༉were larger (20 [17–23] vs. 17 [16–19], *p* < 0.001 and 42 [39–44] vs. 38 [35–42], *p* < 0.001, respectively), and all these differences are statistically significant (Table 1).

**Table 1:** Clinical and demographic characteristics

Characteristics	All patients (*n* = 404)	TAPSE/PASP > 0.43 (*N* = 244)	TAPSE/PASP ≤ 0.43 (*N* = 160)	*P*-value
Age (years)	66 (55, 72)	64 (55, 72)	69 (57, 73)	0.004
Sex, male (*n*, %)	253 (62.62)	147 (60.25)	106 (66.25)	0.223
Smoking(*n*, %)	183 (45.30)	109 (44.67)	74 (46.25)	0.755
BMI (kg/m^2^)	24.3 (4.1)	24.9 (4.1)	23.4 (4.0)	< 0.001
Heart rate (b.p.m.)	84 (70, 98)	83 (68, 95)	85 (74, 98)	0.030
SBP (mmHg)	135 (119, 148)	139 (124, 153)	132 (116, 144)	< 0.001
DBP (mmHg)	80 (68, 88)	79 (68, 86)	80 (72, 89)	0.166
BSA (m²)	1.7 (1.5, 1.8)	1.8 (1.6, 1.9)	1.7 (1.5, 1.8)	< 0.001
Hypertension (*n*, %)	225 (55.69)	125 (51.23)	100 (62.50)	0.026
Diabetes (*n*, %)	159 (39.36)	93 (38.11)	66 (41.25)	0.528
Atrial fibrillation (*n*, %)	122 (30.20)	62 (25.41)	60 (37.50)	0.010
Dyslipidemia (*n*, %)	156 (38.61)	101 (41.39)	55 (34.38)	0.157
CKD (*n*, %)	124 (30.69)	65 (26.86)	59 (36.88)	0.033
Stroke (*n*, %)	108 (26.73)	82 (33.61)	26 (16.25)	< 0.001
Previous MI (*n*, %)	130 (32.18)	75 (30.74)	55 (34.38)	0.444
Previous PCI (*n*, %)	92 (22.77)	65 (26.64)	27 (16.88)	0.022
Previous CABG (*n*, %)	29 (7.18)	8 (3.28)	21 (13.13)	< 0.001
ACEI/ARB/ARNI (*n*, %)	221 (54.70)	136 (55.74)	85 (53.13)	0.606
Beta blocker (*n*, %)	260 (64.36)	154 (63.11)	106 (66.25)	0.520
Diuretics (*n*, %)	332 (82.18)	192 (78.69)	140 (87.50)	0.024
Calcium channel blocker (*n*, %)	81 (20.05)	58 (23.77)	23 (14.38)	0.021
Aldosterone antagonist(*n*, %)	320 (79.21)	188 (77.05)	132 (82.50)	0.187
Nitrates(*n*, %)	221 (54.70)	124 (50.82)	97 (60.63)	0.053
Statins(*n*, %)	342 (84.65)	212 (86.89)	130 (81.25)	0.124
Aspirin/clopidogrel(*n*, %)	281 (69.55)	169 (69.26)	112 (70.00)	0.875
Anticoagulant(*n*, %)	69 (17.08)	42 (17.21)	27 (16.88)	0.930
SGLT-2i (*n*, %)	174 (43.1)	102 (41.8)	72 (45.0)	0.526
TC (mmol/L)	4.1 (3.5, 4.8)	4.1 (3.5, 4.7)	4.1 (3.5, 5.2)	0.209
TG(mmol/L)	1.3 (1.0, 1.9)	1.4 (1.1, 1.9)	1.1 (0.9, 1.8)	< 0.001
LDL-c(mmol/L)	2.1 (1.7, 2.8)	2.0 (1.8, 2.7)	2.4 (1.7, 3.0)	0.233
FBG(mmol/L)	5.7 (5.0, 6.8)	5.7 (5.2, 7.6)	5.6 (4.7, 6.4)	0.093
HbA1c (%)	6.3 (5.9, 7.3)	6.3 (5.9, 7.2)	6.2 (5.9, 7.7)	0.363
Hemoglobin (g/L)	125 (105, 139)	125 (106, 138)	126 (104, 139)	0.958
Hematocrit (%)	37.4 (32.1, 42.7)	37.4 (32.4, 41.6)	37.6 (31.3, 42.7)	0.904
RDW-SD(fL)	44.8 (41.9, 47.6)	44.1 (41.6, 47.2)	45.7 (43.1, 47.9)	0.001
CTnI (ng/mL)	0.03 (0.02, 0.10)	0.02 (0.02, 0.07)	0.03 (0.02, 0.12)	0.008
NT-proBNP(pg/mL)	2690 (1462, 6408)	1810 (1189, 4020)	6290 (2309, 10955)	< 0.001
Serum albumin (g/L)	37.9 (34.8, 40.8)	39.0 (35.2, 41.4)	36.8 (34.3, 39.5)	< 0.001
BUN (mmol/L)	8.3 (5.9, 14.0)	8.2 (6.0, 11.5)	8.3 (5.9, 16.0)	0.370
Creatinine(µmol/L)	90.5 (67.3, 159.6)	93.8 (69.2, 158.5)	79.9 (66.9, 215.3)	0.599
eGFR (mL/min/1.73 m^2^)	63.4 (33.2, 83.3)	60.9 (35.6, 82.1)	67.1 (23.9, 90.1)	0.664
Serumuric acid (µmol/L)	396.3 (319.5,520.3)	389.6 (313.6,516.4)	407.4 (333.7, 538.8)	0.199
Interleukin-6 (pg/mL)	18.2 (11.4)	11.7 (8.0)	28.2 (8.2)	< 0.001
LVIDD (cm)	5.3 (4.9, 5.7)	5.2 (4.8, 5.7)	5.4 (5.0, 5.8)	0.078
LVM (g)	116.7 (99.5, 134.4)	116.6 (98.0, 136.5)	116.7 (105.0, 132.0)	0.222
E/e´	21.7 (16.4, 29.0)	18.5 (15.9, 24.9)	26.1 (18.4, 36.0)	< 0.001
LAD (cm)	4.5 (4.1, 5.0)	4.5 (4.1, 4.9)	4.6 (4.1, 5.1)	0.010
SV (mL)	75 (64, 87)	75 (66, 87)	77 (63, 89)	0.628
CO (L/min)	5.4 (4.8, 6.7)	5.4 (4.8, 6.7)	5.4 (5.1, 6.7)	0.524
LVEF (%)	58 (51, 66)	61 (52, 67)	57 (51, 63)	0.016
IVC (mm)	18 (16, 21)	17 (16, 19)	20 (17, 23)	< 0.001
RAD (mm)	40 (36, 43)	38 (35, 42)	42 (39, 44)	< 0.001

Data are expressed as mean (standard deviation) or as median (Q1, Q3) or as number (percentage)

*ACEI* angiotensin-converting enzyme inhibitors, *ARB* angiotensin receptor blocker, *ARNI* angiotensin receptor neprilysin inhibitor, *BMI* body mass index, *BSA* body surface area, *BUN* blood urea nitrogen, *CABG* coronary artery bypass grafting, *CAD* coronary artery disease, *CKD* chronic kidney disease, *CO* cardiac output, *cTnI* cardiac troponin I, *DBP* diastolic blood pressure, *E/e′*the ratio of mitral peak velocity of early filling E to the velocity of mitral annulus early diastolic motion e′, *eGFR* estimated glomerular filtration rate, *FBG* fasting blood glucose, *IQR* interquartile range, *IVC* inferior vena cava, *IVS* interventricular septum, *LAD* left atrial anterior-posterior dimension, *LDL-C* low-density lipoprotein cholesterol, *LVIDD* left ventricular internal dimension diastole, *LVEF* left ventricular ejection fraction, *LVM* left ventricular mass, *LVPW* left ventricular posterior wall, *MI* myocardial infarction, *NT-proBNP* N-terminal pro-B-type natriuretic peptide, *PASP* pulmonary artery systolic pressure, *PCI* percutaneous coronary intervention, *RAD* right atrium diameter, *RDW-SD* red blood cell distribution width-standard deviation, *SBP* systolic blood pressure, *SGLT-2i* sodium-glucose cotransporter 2 inhibitor, *SV* stroke volume, *TAPSE* tricuspid annular plane systolic excursion, *TC* total cholesterol, *TG* triglyceride

### Association of RV-Arterial uncoupling with clinical variables

Univariate analysis was performed with all of the variables listed above, and those with statistically significant differences were included in the multivariate logistic regression analysis, including age, BMI, hypertension, atrial fibrillation, renal dysfunction, previous percutaneous coronary intervention (PCI), previous CABG, use of diuretics, use of calcium channel blockers, total cholesterol, fasting blood glucose, N-terminal pro-B-type natriuretic peptide, interleukin-6, RDW-SD, serum albumin level, E/e´, LVEF, IVC, left atrium dimensions, and right atrium dimensions. The results of the multivariate logistic regression analysis indicated that E/e´, LVEF, IVC, history of hypertension, atrial fibrillation, postoperative CABG, BMI, interleukin-6, RDW-SD, and serum albumin levels were independent factors influencing RV-arterial uncoupling (all *P* < 0.05, Table 2).

**Table 2:** Multivariate logistic regression analysis of RV-arterial uncoupling

Variable	OR value	95% CI	*P*-value
E/e´	1.04	1.00-1.09	0.045
LVEF	0.85	0.78–0.92	< 0.001
IVC	1.18	1.03–1.38	0.018
Hypertension	6.77	1.96–28.14	0.004
Atrial fibrillation	15.29	4.19–68.60	< 0.001
CABG	31.73	4.55-275.42	< 0.001
BMI	0.77	0.64–0.90	0.002
Interleukin-6	1.51	1.35–1.75	< 0.001
RDW-SD	0.87	0.77–0.97	0.013
Serum albumin	0.74	0.61–0.88	0.001

Values are shown as geometric mean (95% confidence interval)

Odds ratios were calculated using multivariate logistic regression after adjusting for age, prior PCI, renal dysfunction, diuretics use, calcium channel blocker use, total cholesterol, fasting blood glucose, N-terminal pro-B-type natriuretic peptide, left atrium dimension and right atrium dimension. *OR* odds ratios, *E/e´* the ratio of mitral peak velocity of early filling E to the velocity of mitral annulus early diastolic motion e′, *LVEF* left ventricular ejection fraction, *IVC* inferior vena cava, *CABG* coronary artery bypass grafting, *BMI* body mass index, *RDW-SD* red blood cell distribution width-standard deviation

### Combination of 9 Common Indexes

**Fig. 4:**
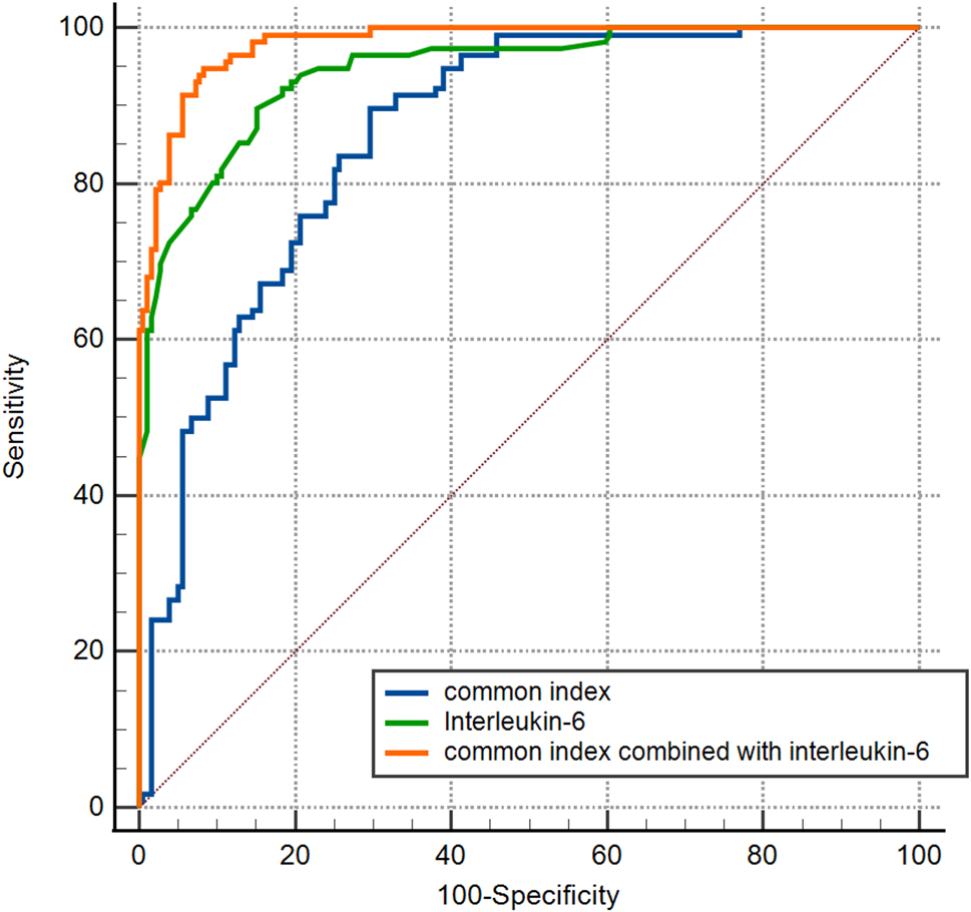
ROC curves of the common index, Interleukin-6, and the common index combined with interleukin-6 for predicting RV-arterial uncoupling. ROC, receiver operating characteristic

A combined diagnostic logistic model using the following 9 common indexes: E/e´, LVEF, IVC, history of hypertension, atrial fibrillation, postoperative CABG, BMI, RDW-SD, and serum albumin, was constructed to obtain the predictive value (probability P1) of the common index. The common index was then combined with interleukin-6 to obtain the common index combined with interleukin-6 predictive value (probability P2), and the ROC curve was plotted together with interleukin-6 (Fig. 4).

### ROC curve analysis of the common index, interleukin-6, and the common index combined with interleukin-6

ROC curve analyses were constructed to evaluate the predictive values of the common index, interleukin-6, and the common index combined with interleukin-6 for the identification of RV-arterial uncoupling. The AUCs for the common index, interleukin-6, and common index combined with interleukin-6 in diagnosing RV-arterial uncoupling were 0.862 (95% CI, 0.817–0.899), 0.944 (95% CI, 0.917–0.965), and 0.980 (95% CI, 0.957–0.993), respectively, with all P values less than 0.001. The test results for the performance of common index, interleukin-6, and common index combined with interleukin-6 in differentiating RV-arterial uncoupling are shown in Table 3. By comparing the diagnostic efficiencies on RV-arterial uncoupling among the common index, interleukin-6, and the common index combined with interleukin-6, it was found that each P value was < 0.001 (Table 4). Among them, the common index combined with interleukin-6 had the highest diagnostic efficiency on RV-arterial uncoupling, with a sensitivity of 94.8% (95% CI 89.1–98.1%), a specificity of 91.6% (95% CI 86.6–95.2%), and an accuracy of 92.9% (95% CI 87.6–96.3%).

**Table 3:** Performance of the common index, interleukin-6, and the common index combined with interleukin-6 in identifying RV-arterial uncoupling

	Sensitivity	Specificity	NPV	PPV	Likelihood ratio (+)	Likelihood ratio (-)	Accuracy
Common index	89.7% (82.6–94.5%)	70.4% (63.1–77.0%)	91.3% (85.9–94.8%)	66.2% (60.8–71.3%)	3.03 (2.40–3.83)	0.15 (0.085–0.25)	78.0% (70.8–83.9%)
Interleukin-6	88.8% (82.8–93.2%)	86.5% (81.5–90.5%)	92.1% (88.3–94.8%)	81.1% (75.7–85.6%)	6.56 (4.76–9.06)	0.13 (0.084-0.20)	87.4% (82.0-91.6%)
Common index combined with interleukin-6	94.8% (89.1–98.1%)	91.6% (86.6–95.2%)	96.5% (92.6–98.4%)	88.0% (81.8–92.3%)	11.32 (6.96–18.40)	0.056 (0.026–0.12)	92.9% (87.6–96.3%)

Values are shown as geometric mean (95% confidence interval)

Common index is a combination of 9 common indexes. *NPV* negative predictive value, *PPV* positive predictive value

**Table 4:** Comparison of diagnostic performance of ROC curves

	Common index Interleukin-6	Common index Common index combined with interleukin-6	Interleukin-6 Common index combined with interleukin-6
Area difference under the curve	0.083	0.119	0.035
Standard error	0.025	0.020	0.010
95%CI	0.034–0.133	0.080–0.157	0.016–0.054
Z value	3.324	6.061	3.648
*P* value	< 0.001	< 0.001	< 0.001

Values are shown as geometric mean (95% confidence interval)

Common index is a combination of 9 common indexes. *ROC* receiver operating characteristic

## Discussion

RV-arterial uncoupling is common in HFpEF and is caused by an impairment of RV intrinsic systolic function and an afterload mismatch. In our previous study [15], it was demonstrated that RV-arterial uncoupling was closely associated with poor outcomes among acute HFpEF patients with CAD, and that the TAPSE/PASP ratio had independent prognostic value. Therefore, identifying risk factors for RV-arterial uncoupling allows for targeted clinical interventions aimed at improving uncoupling status of the RV-arterial system and achieving better treatment outcomes [16].

In our study, the results indicated that a history of hypertension, atrial fibrillation, E/e ´, IVC, postoperative CABG, and interleukin-6 were independent risk factors for RV-arterial uncoupling. Conversely, LVEF, BMI, RDW-SD, and serum albumin levels were protective factors for RV-arterial uncoupling. In patients with HFpEF, although the global LVEF is preserved, there is a deficit in local left ventricular systolic function[17]. Both ventricles share myocardial fibers and a ventricular septum, attributing approximately 20% to 40% of RV systolic function to left ventricular systolic function[18]. Moreover, this study identifies LVEF as a protective factor, indicating that left ventricular systolic function leads to a change in the RV-arterial coupling state by affecting RV systolic function.

Another important factor in the RV-arterial coupling system is the increase in pulmonary artery pressure. In the development of HFpEF, left ventricular diastolic dysfunction and decreased left atrial compliance lead to increased pulmonary artery pressure due to the retrograde transmission of pressure load to the pulmonary venous system [19]. The prevalence of pulmonary hypertension can vary greatly between HFpEF populations of different etiologies [20–22]. In our study, all patients included were CAD patients, except for those who suffered from conditions affecting the change of pulmonary artery pressure, such as CTEPH, COPD, and CKD 5.

In our study, interleukin-6 significantly impacted RV-arterial uncoupling, with an area under the ROC curve of 0.944 (95%CI 0.917–0.965), a sensitivity of 88.75%, and a specificity of 86.48%. Interleukin-6 is involved in the occurrence and development of pulmonary hypertension through signal transduction pathways related to inflammatory responses [23]. Several studies have demonstrated that interleukin-6 is independently correlated with RV function and RV-arterial coupling in patients with pulmonary hypertension [24]. However, the association between interleukin-6 and RV-arterial coupling has rarely been explored in the prevalent CAD population, especially in patients suffering from HF. The accumulation of epicardial adipose tissues is one of the pathophysiological mechanisms of HFpEF occurrence and development [25, 26]. When inflammation occurs, such as with HFpEF comorbidities like hypertension, diabetes, atrial fibrillation and obesity, the epicardium becomes the site of adipogenesis disorders, thereby secreting pro-inflammatory adipocytokines [25]. For example, interleukin-6 and tumor necrosis factor-alpha (TNF-α) act on endothelial cells of the coronary microcirculation, producing reactive oxygen species (ROS). This reduces the bioavailability of nitric oxide (NO) and the activity of soluble guanylate cyclase (sGC) in cardiomyocytes [17]. Consequently, the signal transduction pathway involving cyclic guanosine monophosphate (cGMP) and protein kinase G (PKG) is inhibited, which ultimately leads to the left and right ventricle myocardial remodeling and the release of transforming growth factor β [17]. This results in collagen deposition in the extracellular space, causing atrial and ventricular fibrosis and pulmonary vascular disease [27], ultimately leading to RV-arterial uncoupling. Interleukin-6 and other cytokines have been shown to mediate this process. These findings were confirmed in our study, which also revealed that interleukin-6 may be the substantial, intrinsic cause of the effects of hypertension and atrial fibrillation on RV-arterial uncoupling.

Atrial fibrillation is also common in HFpEF. Several studies have shown a strong link between atrial fibrillation and the presence of RVD in patients with HFpEF [28]. Patients with atrial fibrillation have more severe pulmonary vascular dysfunction and consequent impairment of RV function than patients with HFpEF in sinus rhythm. The presence of atrial fibrillation has been associated with right atrial dilation and a higher right atrial pressure compared to patients who have pulmonary hypertension without atrial fibrillation, accelerating the onset of uncoupling. Currently, scholars have different views on the effect of obesity on the right ventricle and its afterload in patients with HFpEF. Our findings suggest that BMI is a protective factor for RV-arterial uncoupling. The “obesity paradox” was proposed by Alhamshari et al. [29] in 2017. This paradox claims that after acute myocardial infarction, the RV function estimated by TAPSE in obese patients would be better than that in non-obese patients. The BMI should be divided into two parts: lean body mass (LBM) and fat mass (FM) [30]. Konishi M et al. claimed that lower values of both muscle and FM were associated with higher mortality in HF [31]. This may be why the BMI appears as a protective factor in our study. TAPSE may be significantly reduced in patients undergoing CABG and/or valve replacement [32]. Our findings suggest that postoperative CABG is an independent risk factor for the development of RV-arterial uncoupling. The significant reduction in TAPSE may be mediated by pericardial adhesions after pericardial resection [17]. Other potential mechanisms include cytokine release, RV infarction due to ischemia or air emboli, and postoperative inflammation or effusion [32]. An elevated IVC internal diameter indicates volume overload, and E/e´>14 indicates increased left atrial pressure. This reflects hemodynamic abnormalities and volume overload can lead to cardiac decompensation and deteriorated RV function. Serum albumin levels are valuable in protecting endothelial function and preventing vascular injury [33], further improving the prognosis of cardiac mortality in patients with HF [34, 35]. This may be the basis of its effect on pulmonary artery endothelial function. Therefore, serum albumin acts as a protective factor for RV-arterial uncoupling regarding nutritional status improvement and inflammation.

This study has several limitations. First, beyond interleukin-6, the effects of various inflammatory cytokines on the RV-arterial coupling system warrant further investigation. Second, this study does not distinguish between LBM and FM in BMI. The protective role of BMI and the influence of its components (LBM vs. FM) on RV-arterial uncoupling require validation in large-sample trials to confirm their correlation with TAPSE/PASP. Third, given that invasive Ees/Ea based on the right heart catheterization is the gold standard for RV-arterial coupling, non-invasive method TAPSE/PASP needs future validation by direct invasive measurement. Fourth, since some patients from whom PASP data were missing were excluded, there may be a selection bias in sampling. Fifth, due to the cross-sectional nature of this study, a causal relationship cannot be established. However, the findings suggest that interleukin-6 may serve as a strong associative biomarker. Furthermore, the absence of an independent validation cohort limits the generalizability and clinical applicability of the proposed common index and interleukin-6 cutoff values, pending validation in larger, prospective studies. Finally, our study is a single-center study with a relatively small sample size and the findings need to be validated in a multicenter study in the future.

In conclusion, among acute HFpEF patients with CAD, a history of hypertension, atrial fibrillation, CABG, interleukin-6, IVC inner diameter, and E/e ´ are independent risk factors for RV-arterial uncoupling. BMI, RDW-SD, serum albumin level, and LVEF are protective factors. Among these factors, interleukin-6 had the greatest effect on RV-arterial uncoupling and the highest evaluation efficiency. All of the above-mentioned influencing factors may be used as therapeutic targets to improve RV-arterial uncoupling. The clinical value of interleukin-6 is especially worth additional attention.

## Data Availability

The datasets generated and analyzed during the current study are available from the corresponding author on reasonable request.
